# Bempedoic acid within the phase ternary diagram governing biliary cholesterol solubilization and gallstone risk

**DOI:** 10.1016/j.jlr.2026.101056

**Published:** 2026-05-12

**Authors:** Agostino Di Ciaula, Mohamad Khalil, Gabriella Garruti, Piero Portincasa

**Affiliations:** 1 Clinica Medica ‘A. Murri’, Department of Precision and Regenerative Medicine and Ionian Area (DiMePre-J), University of Bari ‘Aldo Moro’, Bari, Italy; 2 Section of Section of Endocrinology, Andrology and Metabolic Diseases, Department of Precision and Regenerative Medicine and Ionian Area (DiMePre-J), University of Bari ‘Aldo Moro’, Bari, Italy

**Keywords:** bempedoic acid, gallstones, bile, bile acid, cholesterol, lithogenicity

## Abstract

Bempedoic acid has a powerful hypocholesterolemic activity and decreases the risk of major adverse cardiovascular events. However, results from the CLEAR Outcomes trial documented an increased incidence of gallstone disease, with mechanisms still unexplained. This evidence generates critical concerns in a worldwide scenario characterized by an increasing incidence of cholelithiasis and by an increasing prescribing trend of bempedoic acid. Therapy with bempedoic acid affects key pathways involved in cholesterol/bile acids secretion and in bile acid homeostasis, possibly leading to cholesterol gallstone formation. Bempedoic acid can increase the expression of *ABCG5* and *ABCG8* genes leading to increased cholesterol efflux, can inhibit the expression of the *Cyp7a1* gene, leading to decreased bile acid synthesis (secondary to decreased Cyp7a1 mRNA and protein), and can inhibit OATP1B1 and OATP1B3 transporters, leading to reduced uptake of bile acids from the entero-hepatic circulation. Bempedoic acid can also inhibit liver MRP1 transporter and glutathione efflux into blood, can increase nuclear FXR transcriptional activity and can suppress PXR activation, with multi-level effects on bile acid/lipid homeostasis and bile lithogenicity, as anticipated by the analysis of the ternary cholesterol–phospholipid–bile salt phase diagram. The risk of cholesterol crystallization and lithogenesis increases on the background of well-known predisposing conditions such as advanced age, female gender, obesity, associated diseases or pharmacological treatments. Future lines of research need to better dissect the interplay between bempedoic acid and the pathogenesis of cholesterol gallstones, particularly in individuals at increased risk for gallstones.

## Graphical Abstract

**Figure:**
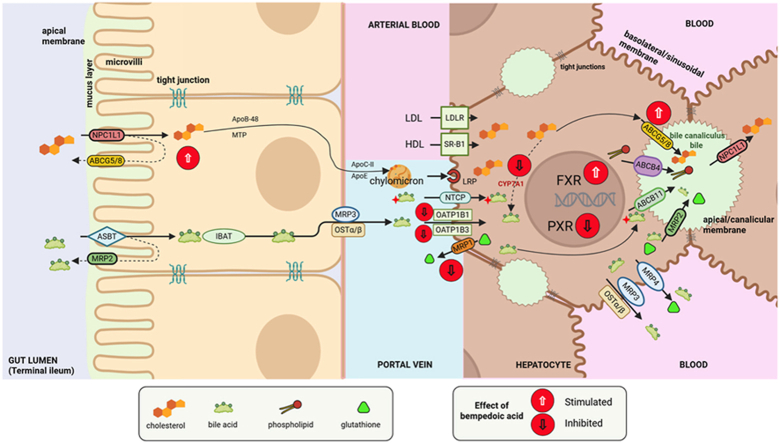

Bempedoic acid (8-Hydroxy-2,2,14,14-tetramethylpentadecanedioic acid, molecular formula C19H36O5, molecular weight 344.5 g/mol) is a new drug with effective hypocholesterolemic activity, either as monotherapy or in combination with existing lipid-lowering therapy in patients at high cardiovascular risk. Bempedoic acid has rapidly gained popularity as a therapeutic tool to treat individuals with statin intolerance and to potentiate the LDL-cholesterol-lowering effects of ezetimibe (1) or statins (2), when LDL-C goals are not achieved with the maximum tolerated dose of a statin (3).

Bempedoic acid is a prodrug requiring enzymatic activation by the very-long chain acyl–CoA synthetase 1 (ACSVL1, encoded by the *SLC27A2* gene), which is almost exclusively expressed in the liver. The dicarboxylic acid is conjugated with coenzyme A (CoA) to form the pharmacologically active thioester (bempedoic acid–CoA). At this level, bempedoic acid is a potent and selective inhibitor of ATP citrate lyase (ACLY), a key enzyme in the biosynthetic pathway of cholesterol and fatty acids (4) (Fig. 1). Bempedoic acid and statins share the same metabolic pathway, but on two different enzymatic steps (5), since bempedoic acid reduces the production of acetyl-CoA, thereby decreasing the downstream synthesis of HMG-CoA, mevalonate, squalene, and ultimately cholesterol in hepatocytes. This mechanism occurs upstream of the target of statins, which inhibit HMG-CoA reductase (6). An additional mechanism involves the enhanced hepatic LDL uptake through increased LDL receptor expression (4). Due to its powerful hypocholesterolemic activity, treatment with bempedoic acid has been associated with a lower risk of major adverse cardiovascular events (7).

**Fig. 1:**
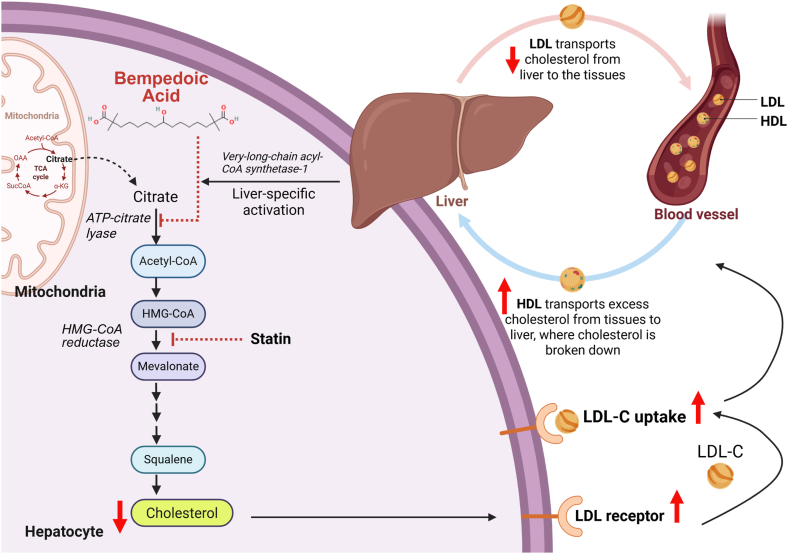
Mechanism of action of bempedoic acid in hepatic cholesterol synthesis and lipoprotein metabolism. Bempedoic acid is a prodrug that is selectively activated in the liver by very-long-chain acyl-CoA synthetase-1 (ACSVL1). The active metabolite inhibits ATP-citrate lyase (ACLY), an upstream enzyme in the cholesterol biosynthesis pathway that converts citrate to acetyl-CoA. Inhibition of ACLY reduces the production of acetyl-CoA, thereby decreasing downstream synthesis of HMG-CoA, mevalonate, squalene, and ultimately cholesterol in hepatocytes. This mechanism occurs upstream of the target of statins, which inhibit HMG-CoA reductase. Reduced hepatic cholesterol synthesis promotes upregulation of low-density lipoprotein (LDL) receptors on hepatocyte membranes, increasing LDL-cholesterol (LDL-C) uptake from the circulation and lowering plasma LDL-C levels. In the circulation, LDL transports cholesterol from the liver to peripheral tissues, whereas high density lipoprotein HDL mediates reverse cholesterol transport from tissues back to the liver for metabolism and clearance. Abbreviations: ACLY, ATP-citrate lyase; ACSVL1, very-long-chain acyl-CoA synthetase-1; Acetyl-CoA, acetyl coenzyme A; HDL, high-density lipoprotein; HMG-CoA, 3-hydroxy-3-methylglutaryl coenzyme A; LDL, low-density lipoprotein; LDL-C, low-density lipoprotein cholesterol; TCA cycle, tricarboxylic acid cycle. Created in https://BioRender.com.

Although long-term therapy with bempedoic acid is generally well tolerated, the CLEAR Outcomes trials (7, 8) revealed a low-grade and reversible increase in serum uric acid and creatinine in the bempedoic acid group, as compared with the placebo group. This finding derives from the competition for the Organic Anion Transporter 2 (OAT2) in the renal tubules (7).

Besides this evidence, the same trial also during the median duration of follow-up (40.6 months) documented an increased incidence of gallstones in the bempedoic acid group, as compared with the placebo group (2.2% vs. 1.2%, respectively) (7, 8). The estimated exposure-adjusted incidence rate was 0.6 and 0.3 per 100 patient years for bempedoic acid and placebo, respectively (9). The CLEAR Outcomes study included patients at high risk for cardiovascular disease, and, therefore, it is not representative of the general population. Nevertheless, the gallstone incidence rate recorded in the bempedoic group but not in the placebo group seems higher than that estimated by a recent systematic review and meta-analysis based on 115 selected studies with 32,610,568 participants. In particular, 6% of the population have gallstones globally (i.e., prevalence) while, according to 12 studies, the incidence of gallstones was 0.47 per 100 person-years [95% CI, 0.37–0.51] without differences between males and females, and with increasing incidence in more recent studies (10).

At variance with the effects of bempedoic acid on serum uric acid and creatinine, the potential increased risk of gallstone formation has not been clearly explained, but offers several points for discussion. The reported initial evidence would generate concerns in a worldwide scenario characterized by the global increasing trends of overweight and obesity (11), metabolic syndrome and associated conditions (12), including metabolic-dysfunction associated steatotic liver disease (MASLD) (13), and cholesterol cholelithiasis (10). In addition, there are increasing prescribing trends for bempedoic acid, as reported by NHS England in the UK, with costs increasing from 484,094 £ in January 2024 to 1,489,720£ in January 2025 (https://opeprescribing.net/chemical/0212000AK/).

In this scenario, addressing potential pathophysiologically relevant mechanisms linking therapy with bempedoic acid with the pathogenesis of gallstones can pave the way to prevention strategies and to a better use of a precious pharmacological resource.

## Biochemical Pathophysiology of Cholesterol Lithogenesis

**Fig. 2:**
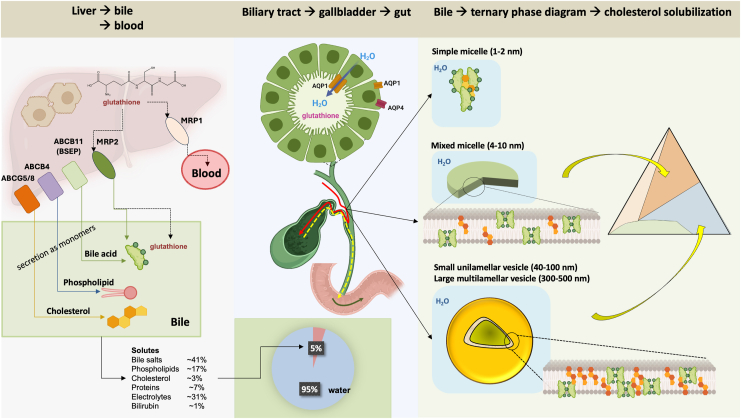
Physical states of lipids in human bile. The levels of liver, biliary tract, and bile are depicted, where the aggregation of the three biliary lipids (i.e., bile acids, cholesterol, and phospholipids) occurs as simple/mixed micelles and small/unilamellar vesicles. In this scenario, the secreted cholesterol in bile is totally kept in solution by the two other two physiological lipid carriers. See text for explanation. Abbreviations: ABCG5/8, ATP-binding cassette subfamily G member five-eighths; ABCB4, ATP-binding cassette subfamily B member 4; MRP1, Multidrug Resistance-associated Protein 1; MRP2, Multidrug Resistance-associated Protein 2; ABCB11, ATP-binding cassette subfamily G member 11; BSEP, Bile Salt Export Pump; AQP, aquaporin. Created in https://BioRender.com.

Human bile is about 95% water and is the only route for the excretion of body cholesterol. Since cholesterol is virtually insoluble in water, the two other biliary lipids act as physiological carriers i.e., bile acids and phospholipids. Both cholesterol and phospholipids are highly insoluble in water and for this they must aggregate with bile acids to shape specific cholesterol carriers, that is, micelles and vesicles. As shown in Figure 2, bile acids, phospholipids (95% as phosphatidylcholine), and free (unesterified) cholesterol are the three lipids which are actively secreted from the liver across the canalicular membrane into the bile canaliculus by specific transporters which requires ATP-dependent transport. Bile acids are the main organic solute contained in bile (concentrations of 20–30 mmol/L), and are the primary force of the bile acid-dependent flow (about 75% of biliary flow), as compared to the 25% of the bile acid-independent flow, i.e., the estimated flow when bile acids are not secreted, dependent on hepatic and cholangiocyte secretion. Bile acids use the ATP-binding cassette (ABC) ABCB11 (also named bile salt export pump, BSEP), and this is the rate limiting step in the enterohepatic recirculation of bile acids. Both newly synthesized and recirculated conjugated bile acids are transported into the bile canalicular space against a steep concentration gradient. The affinity of ABC11 is high for conjugated bile acids, but low for unconjugated bile acids which are poorly transported across this pathway.

Phospholipids use the ABCB4 transporter, while cholesterol uses the ABCG5/G8 transporter (14, 15).

**Fig. 3:**
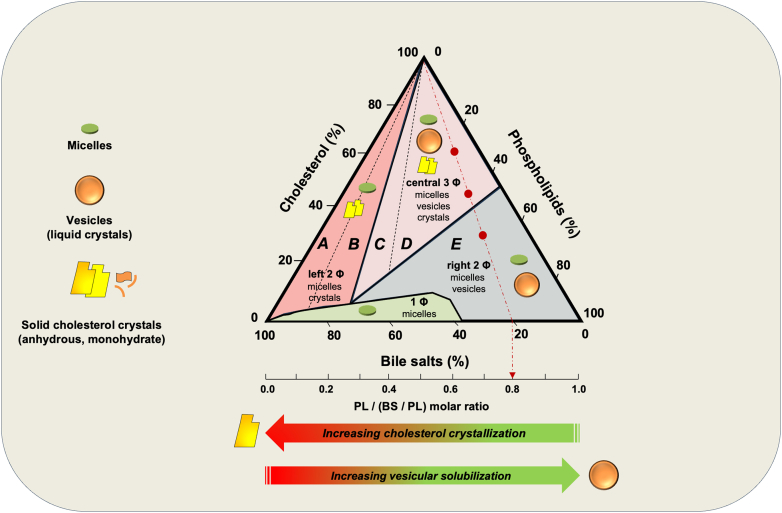
The equilibrium ternary phase diagram and biliary cholesterol solubility. Concentrations of the three biliary lipids cholesterol, phospholipids and bile salts, are expressed in moles percent ranging from 0% to 100%. Based on the solid and liquid crystallization sequences present in the bile *at equilibrium*, the green 1-phase (θ) zone at the bottom of the triangle contains only micelles (micellar zone). Above, the three zones are the left 2 θ with regions A, B enriched in saturated micelles and solid cholesterol monohydrate crystals, the central 3 θ with regions C, D which enriched in saturated micelles, vesicles (liquid crystals), and solid cholesterol monohydrate crystals. The right 2 θ with region E is enriched in saturated micelles and vesicles. At the bottom of the diagram, the horizontal line depicts the phospholipid (PL)/(bile salt (BS) + PL ratio), and any line connecting the bottom axis with the top of the triangle (100% cholesterol) plots an identical PL/(BS/PL) ratio. As an example in the figure, all biles plotting on the coarse interrupted red line exhibit an identical ratio of 0.8, although the relative amounts of cholesterol increase when moving from bottom to top. The two arrows at the bottom of the triangle point to opposite pathways of cholesterol presence in bile in relation to zones and regions. See text for explanation. Adapted from Wang and Carey (19) and Portincasa and Wang (20, 21, 22, 23).

The bile acid–independent flow requires the secretion of glutathione, glutathione conjugates, and organic anions by specific transporters on the canalicular membrane. Glutathione is found in the hepatocyte cytosol (5–10 mmol/L) and is exported into bile via another ABC transporter, the multidrug resistance-associated protein 2 (MRP2), which also transports unconjugated and dianionic/sulphated conjugated bile salts (16), and glucuronidated conjugates of drugs, toxins, bilirubin, and bicarbonate (HCO_3_-). The multidrug resistance-associated protein 1 (MRP1) functions as a transporter of glutathione into the blood. In the biliary tract, the membrane protein aquaporin 1 (AQP1) in human and rodent cholangiocytes is responsible for the apical secretion of water during both basal- and hormone-regulated ductal bile formation. The cells lining bile ducts have the AQP1 and AQP4 and at their basolateral membranes (17, 18) (Fig. 2). During the bile flow, bile acids remain monomeric up to their critical micellar concentration which is 1–3 mM but at higher concentration bile acid monomers self-aggregate as simple micelles (size 1–2 nm), i.e., shaped like disks which bind a molecule of cholesterol and increase its aqueous solubility. No phospholipids appear in simple micelles. Simple micelles can grow to larger mixed micelles (size 4–10 nm) which incorporate also phospholipids and at least three times more cholesterol, compared to simple micelles. In normal bile, simple bile acid micelles and mixed bile acid-lecithin micelles co-exist in a ratio of 1:5. Mixed micelles are lipid bilayers with the hydrophilic groups of the bile acids and phospholipids aligned on the outside of the bilayer and the hydrophobic groups on the inside with cholesterol molecules solubilized on the inside of the bilayer away from the aqueous areas on the outside. Notably, the amount of solubilized cholesterol depends on the relative proportions of bile acids, with maximal solubility occurring when the molar ratio of phospholipids to bile acids is between 0.2 and 0.3 (see in particular the ternary phase diagram of Fig. 3). As further depicted in Figure 2, phospholipids in an aqueous environment can self-aggregate to form stable bilayer vesicles containing also a trace amount of bile acids, if any, and more cholesterol molecules in between the hydrophobic acyl chains of phospholipids. Unilamellar vesicles appear as larger spherical structures in which even more cholesterol is solubilized into the bilayers of phospholipids. The ratio of unilamellar vesicles to micelles depends on the bile acid and cholesterol concentrations of the bile, i.e., is greatest in bile with low bile acid and high cholesterol concentrations. In addition, at low bile acid concentrations, the biliary lipids often aggregate within large unilamellar (∼40–100 nm in diameter) or multilamellar (∼300–500 nm in diameter) vesicles. Bile acids at high concentrations can dissolve vesicles to form small mixed micelles (∼4–8 nm in diameter) (14).

**Fig. 4:**
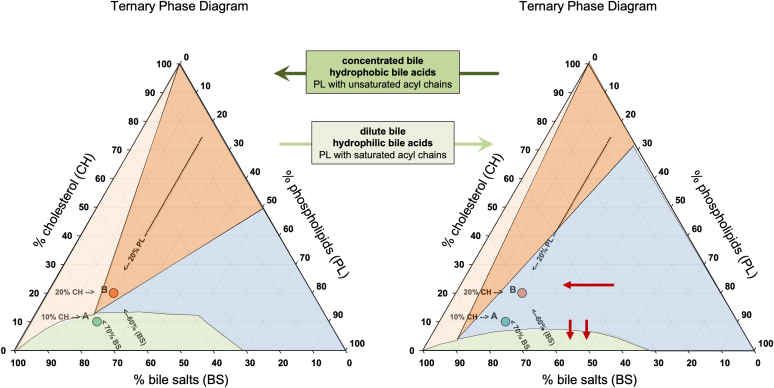
Dynamic shift of zones within the ternary phase diagram as a consequence of change of molar concentrations of biliary lipids, bile dilution, bile acid hydrophilicity, and phospholipid composition. The left panel refers to a standard cholesterol–phospholipid (lecithin)–mixed bile salt system with a total lipid concentration of 10.5 g/dl (26, 27) *at equilibrium*. Note the large micellar area and the large left 2-phase zone and the central 3-phase zone, at the expenses of the right 2-phase zone. The green point A plots in the micellar zone, while the red point B plots within the central supersaturated 3-phase zone containing supersaturated micelles, vesicles and also cholesterol crystals. In the right panel the total lipid concentration decreases to 3.5 g/dl. The red arrow indicate a leftward shift of the right 2-phase (micellar, vesicular) zone which enlarges to at the expense of crystal-containing zones, while the one-phase micellar zone at bottom is markedly reduced. In this case the shift is associated with the re-positioning of points within the right 2-phase zone where cholesterol crystals are absent (see text for explanation).

If hepatic cholesterol output exceeds its solubilizing capacity, bile becomes supersaturated with cholesterol (24), and excess cholesterol cannot be adequately solubilized into simple/mixed micelles and vesicles *at equilibrium*. The physical state of lipids in human bile is clearly shown in the ternary phase diagram where the relative concentrations of cholesterol, phospholipids and bile salts plot as moles percent within different phasic zones and regions (25) (Figs. 3 and 4). The diagram depicted in Figure 3 refers to a standard cholesterol–phospholipid (lecithin)–mixed bile salt system at 37 °C, 0.15 M NaCl, pH 7.0, and a total lipid concentration of 7.5 g/dl (26, 27) *at equilibrium*. The top corner of the triangle indicates 100% cholesterol, the left bottom corner 100% bile salts, and the right bottom corner 100% phospholipid. Any point within the triangle plots at the intersection of each molar concentration of cholesterol, phospholipids, and bile salts, and predicts whether cholesterol is dissolved in bile into vesicle, vesicles, or is nucleating as solid anhydrous and then monohydrate crystals *at equilibrium* (28, 29). The relative proportions of the three lipids in bile play a critical role in determining the maximal solubility of cholesterol, and the three components can aggregate as different phases within different zones. Based on the solid and liquid crystallization sequences present in the bile, the 1-phase (θ) zone contains micelles (micellar zone). Above, the left 2 θ zone containing regions A, B are enriched in saturated micelles and solid cholesterol monohydrate crystals, while the central 3 θ zone with regions C, D is enriched in saturated micelles, vesicles (liquid crystals), and solid cholesterol monohydrate crystals. The right 2 θ zone (region E) is enriched in saturated micelles and vesicles. At the bottom of Figure 3, the horizontal line depicts the phospholipids (PL)/(bile salts (BS)/PL) molar ratios ranging from 0.0 (left) to 1.0 (right). Any line connecting the bottom axis with the top of the triangle (100% cholesterol) plots an identical PL/(BS/PL) ratio, with increased relative amounts of cholesterol when moving from bottom to top. As an example, in the figure all biles plotting on the coarse interrupted red line exhibit an identical ratio of 0.8.

Manipulation of bile content will greatly affect the characteristics of the phase diagram and crystallization capacity. Within the 1-phase micellar zone, bile appears as a visually clear stable solution, and is termed “unsaturated” and capable of solubilizing cholesterol. At the boundary line of the micellar zone, because of higher cholesterol concentrations, bile becomes “saturated” because the solubilizing capacity for cholesterol is maximized by micelles. Thus, cholesterol appears in more than one phase within each zone moving from A to E, i.e., micelles, vesicles, and solid cholesterol crystals. In particular, cholesterol precipitation and crystallization can increase at increasing concentrations of cholesterol in the central and left zones, above the micellar zone. Cholesterol precipitation is also rapid in case of defective bile acids, whereas at increasing amounts of phospholipids, cholesterol may reside in vesicles with phospholipids. At this moment solid cholesterol crystal formation is slower or absent.

In conclusion, a prerequisite for cholesterol lithogenesis (30) is when in the ternary phase diagram a given bile plots above the safe unsaturated (1-phase, micellar) zone or above the right (2-phase, micellar, vesicular) *E* zone (19, 20, 31, 32).

Importantly, the ternary phase diagram is dynamic in its nature *at equilibrium,* as shown in Figure 4 (between left and right panel). Indeed, the crystallization pathways can change under several conditions, including bile concentration/dilution, bile acid hydrophilicity/hydrophobicity, type of unsaturated/saturated phospholipids, and temperature. In general, whereas excess cholesterol and bile concentration increase the probability of crystallization from vesicles, excess bile acids, hydrophobic bile acids, phospholipids with unsaturated acyl chains or concentrated bile will promote micellar and vesicular solubilization of cholesterol (33, 34).

Examples include a shift of total lipid concentration from 2.5 (low) to 7.5 (high) g/dl (i.e., hepatic dilute bile vs. gallbladder concentrated bile), enrichment in phospholipids with saturated acyl chains (i.e., sphingomyelin), and low temperature (37 °C→4 °C). Concerning the relative hydrophilicity of bile acids, for unconjugated bile acids, the rank is UDCA (3α, 7β, dihydroxy) > CA (3α, 7α, 12α trihydroxy) > CDCA (3α, 7α-dihydroxy) > DCA (3α, 12α dihydroxy) > LCA (3α, monohydroxy). Such rankings remain the same irrespective of the type of conjugation, considering that the hydrophilicities of bile acid conjugation types are taurine conjugates > glycine conjugates > free bile acid.

In Figure 4 in the left panel, the diagram refers to a standard cholesterol–phospholipid (lecithin)–mixed bile salt system at 37 °C, 0.15 M NaCl, pH 7.0, and a total lipid concentration of 10.5 g/dl (26, 27) *at equilibrium*. The two points represent a mixture containing different proportions of cholesterol, phospholipids, and bile salts, each contributing to the maximum value of 100%. If the total lipid concentration (i.e., bile concentration) remains constant, the area belonging to the 1-phase micellar zone at the bottom of the triangle remains enlarged and constant. The left 2-phase (micellar, crystal) zone and the central 3-phase (micellar, vesicular, crystal) zone are quite enlarged, at the expense of the right 2-phase (micellar, vesicular) zone. The green point A represents a mixture of cholesterol:bile salts:phospholipids = 10:70:20 mol % (similar to human unsaturated bile plotting within the green 1-phase micellar zone). By contrast, in cholesterol stone-prone individuals or following the effect of molecules increasing cholesterol efflux from liver into bile the red point B represents a mixture of cholesterol:bile salts:phospholipids = 20:60:20 mol % in which cholesterol concentration increases, bile salt concentration decreases and phospholipid concentration remains constant. In this scenario, point B will lay within the central supersaturated 3-phase zone (in particular areas C-D) where excess cholesterol can nucleate into solid crystals due to insufficient micellar and vesicular solubilization and ultimately aggregate as stones.

A dynamic transformation within the ternary phase diagram is depicted in the right panel of Figure 4 upon decreased total lipid concentration. The example refers to a standard cholesterol–phospholipid (lecithin)–mixed bile salt system at 37 °C, 0.15 M NaCl, pH 7.0, and a total lipid concentration of 3.5 g/dl. Here, all crystallization pathways shift to the left to lower phospholipid contents, the right 2-phase (micellar, vesicular) zone enlarges to the left at the expense of crystal-containing zones, and the one-phase micellar zone at bottom is markedly reduced (arrows). In this context, the time needed to equilibrium state increases progressively, with the result that cholesterol crystallization is much slower, and gallstone formation impeded. In this scenario, both points will be included in the right 2-phase zone containing micelles and vesicles meaning cholesterol still in solubilized. As already mentioned, a similar shift occurs with hydrophilic bile acids, or PL enriched in saturated acyl chains.

Additional pathogenetic pathways for cholesterol gallstones derive from intestinal factors (i.e., increased intestinal cholesterol absorption observed in lithogenic in animal models (35, 36), gut dysbiosis associated with high concentrations of the secondary hydrophobic bile acid deoxycholic acid conjugates), and from gallbladder motor dysfunction and inflammation (37).

Available evidence, mostly deriving from preclinical and experimental studies, suggests that long-term therapy with bempedoic acid could affect several factors, mainly through the effect of this drug on hepatic cholesterol/bile acids secretion, and on bile acid homeostasis. Most important aspects in this complex scenario will be discussed in the following paragraphs, aligning available pathophysiological evidences with the effects of bempedoic acid.

### Effects of bempedoic acid on ABCG5/8 and increased biliary secretion of cholesterol

In the hepatocyte, ATP-binding cassette transporter subfamily G member 5 (ABCG5) and 8 (ABCG8), encoded by the *ABCG5* and *ABCG8* genes, control cholesterol efflux in bile (38). The ABCG5/8 heterodimer complex (39) is the functional transporter of cholesterol at the level of hepatocyte canalicular membrane (40). ABCG5/8, in particular, mediates the excretion of about 82% of hepatic cholesterol into bile, and an upregulation of *ABCG5* and *ABCG8* genes can promote the formation of cholesterol gallstones (39, 41, 42, 43, 44, 45, 46). In the animal model of *Ldlr*^*−/−*^ mice on a high-fat, high-cholesterol diet, supplement of diet with bempedoic acid for 12 weeks increased the expression of *ABCG5* and *ABCG8* (47). Other experiments using mouse or human primary hepatocytes as models showed that treatment with metformin upregulated the expression of *ABCG5/8* through inhibition of ATP citrate lyase (48), the same target of bempedoic acid.

In the enterocyte, the heterodimer of ABCG5 and ABCG8 is an apical sterol export pump promoting active efflux of cholesterol and plant sterols from the enterocytes back into the intestinal lumen for fecal excretion (14). This step, in concert with the sterol efflux transporters ABCG5 and ABCG8 on the canalicular membrane of hepatocytes and ABCB11, a bile acid export pump, plays a crucial role in the regulation of hepatic cholesterol secretion and avoids cholesterol accumulation in the body (14, 49). To date, there is no evidence that ATP citrate lyase inhibition by bempedoic acid can affect intestinal *ABCG5/8* gene expression or activity. However, in a model of transgenic mice that overexpress human *ABCG5* and *ABCG8* in the liver but not the intestine, overexpression of human *ABCG5* and *ABCG8* enhanced hepatobiliary secretion of cholesterol and increased the amount of intestinal cholesterol available for absorption and fecal excretion, but did not reduce fractional intestinal cholesterol absorption (50). In this scenario, the role of the canonical intestinal Niemann-Pick-C1 like-1 protein (NPC1L1) transporter should be taken into account. In fact, in animal models, deletion of the hemitransporter ABCG8 does not affect the acute uptake rates of cholesterol by the small intestine via NPC1L1 (49).

However, although long-term ATP citrate lyase inhibition could increase the cholesterol efflux in bile through an upregulation of *ABCG5* and *ABCG8* genes, it seems unlikely that this pathway can be sufficient per se to increase bile lithogenicity and the risk of cholesterol lithogenesis. This hypothesis is supported by previous evidence on the effect of pravastatin on bile composition. As bempedoic acid, pravastatin is also able to upregulate *ABCG5/ABCG8*, increasing its mRNA expression (51). However, in humans, biliary lipids and cholesterol saturation index are unaffected during 1-month (52) or 3-months therapy (53) with pravastatin. By contrast, after one year of treatment, pravastatin significantly decreases the cholesterol saturation index of gallbladder bile (53) and, in animal models, treatment with pravastatin prevented cholesterol gallstone formation (54).

### Effects of bempedoic acid on bile acid synthesis and transport and decreased secretion of bile acids

The classical pathway contributing to formation of primary bile acids in the liver produces about 90% of the human bile acid pool. The rate-limiting enzyme cytochrome P450 Family 7 Subfamily A Member 1 (CYP7A1) is involved at this level in the endoplasmic reticulum, and catalyzes the transformation of cholesterol to 7α-hydroxycholesterol to regulate the overall amount of bile acid production. The alternative (‘acidic’) pathway, in physiological conditions, produces about 10% of the total synthesis of bile acids in humans, and occurs from cholesterol transported into mitochondria. Also in this case, bile acid synthesis begins with CYP27A1, leading to C27 oxidation of cholesterol to a spectrum of alcohol, aldehyde, and carboxylic acid metabolites. Thus, CYP7A1 is the key rate-limiting enzyme of bile acid synthesis. *Cyp7a1* gene transcription is regulated by bile acids returning to the liver via the enterohepatic circulation (55), and the feedback modulation is primary dependent on the intestinal FXR/FGF19-to-liver FGFR4 signaling pathway (56). In an animal model of liver steatosis, i.e., HFHFr—female Sprague-Dawley rat fed a high-fat high-fructose diet, bempedoic acid decreased fatty liver, but also inhibited the expression of the *Cyp7a1* gene, thus reducing Cyp7a1 protein (57).

Besides bile acid synthesis, the effects of bempedoic acid on bile acid homeostasis also involve a modulatory effect on bile acids transporters. In the hepatocyte, the uptake of bile salts from portal blood flow is mainly due to basolateral uptake transporters such as organic anion transporting polypeptides (OATPs) (58), and Na + /taurocholate cotransporting polypeptide (NTCP) (59). The canalicular excretion in bile is mediated by efflux transporters, such as bile salt export pump (BSEP, also known as ABCB11) (16, 60) and multidrug resistance-associated protein 2 (MRP2) (16). OATPs and Organic Anion Transporters (OATs) are largely expressed in the liver, and OATP1B1, OATP1B3, and OATP2B1 play a critical role in uptake of bile acids within the entero-hepatic circulation (61). OATP1B1, in particular, is encoded by *SLCO1B1* gene. A *SLCO1B1* polymorphism (SLCO1B1 Exon4 C > A) has been associated, in humans, with increased risk for gallstones (62).

Available evidence suggests that bempedoic acid (63) and its glucuronide (64) have a weak inhibitory effect on OATP1B1 and OATP1B3. Although, at the moment, transcriptomic evidence exploring possible effects of bempedoic acid on *SLCO1B1* (encoding OATP1B1) or *SLCO1B3* (encoding OATP1B3) gene expression is lacking, a pharmacological inhibition of *SLCO1B1* gene expression by bempedoic acid and its metabolite, or a post-translational (i.e., functional) direct inhibition of OATP1B1 and OATP1B3 transporters might contribute to increase the risk of cholesterol gallstones.

### Effects of bempedoic acid on glutathione levels and biliary flux

Comprehensive in silico analyses comparing biological activities of major phenolic compounds (oleocanthal, oleacein, luteolin and tyrosol) with ezetimibe and bempedoic acid, revealed that bempedoic acid is a important inhibitor of the human ATP-binding cassette transporter multidrug resistance protein 1 (MRP1) (63). MRP1, encoded by *ABCC1* gene, acts as a primary active transporter of structurally diverse organic anions, mediates the sinusoidal efflux of divalent bile salt conjugates (65), and is involved in a number of glutathione-related cellular processes (66). Although MRP1 is not detectable in the gallbladder (67), MRP1 is located in the hepatocyte on basolateral/sinusoidal membrane and in intracellular vesicles, contributing to efflux into blood of the cellular glutathione S-conjugate, a mechanism protecting against oxidative stress (68). The multispecific organic anion transporter MRP2 (also termed ABCC2), located on the apical/canalicular membrane of hepatocytes, is involved in the ATP-dependent efflux of glutathione into bile (14, 69, 70). To date, there is no evidence of interaction between bempedoic acid and MRP2, and this transporter has an important role in hepatic GSH secretion (14). Notably, bile flow is correlated with either biliary excretion of glutathione and endogenous bile acids (71). In particular, the components of bile flow consist of bile acid-dependent and -independent bile flow. Bile acid-dependent bile flow varies directly with hepatic bile acid output. It is estimated that bile acid-independent bile flow ranges from 1.5 to 2.0 μl/min/kg/body weight in humans. Hepatic secretion of GSH and bicarbonate (HCO3-) are the major components of the bile acid-independent fraction of bile flow. Glutathione is an osmotic driving force in hepatic bile secretion (71), and it has been estimated that 1 μmol of glutathione obligates the excretion of 34 μl of bile (72). Since oxidative stress and inflammatory cytokines can alter bile composition and cholesterol metabolism, they could indirectly influence individual susceptibility to gallstone formation. In mice, lithogenic diet inhibits glutathione output from the liver (73), and decreases activities of hepatic glutathione reductase and glutathione-S-transferase (74). The association of capsaicin, curcumin, or their combination with a lithogenic diet is able to significantly reduce the incidence of cholesterol gallstones, as these nutrients counteract the decreased activities of hepatic glutathione reductase and glutathione-S-transferase caused by the lithogenic diet (74).

Patients with gallstones have lower levels of glutathione and glutathione-related enzymes, catalase and superoxide dismutase than gallstone-free controls, with increased oxidative stress in the gallbladder mucosa (75). A role for defective glutathione hepatic metabolism can also contribute to increased cholesterol lithogenesis (76), as also suggested by our research (77).

The potential interactions between bempedoic acid and MRP1 rely on a single in silico evidence, and direct evidence exploring the effects of bempedoic acid on biliary glutathione and bile flow is lacking. Nevertheless, we might speculate that the inhibitory effect of bempedoic acid on MRP1 (63) affects hepatic bile composition through an increased bile acid-independent bile flow linked with a decreased hepatic secretion of glutathione by MRP1 into blood, and a potential compensatory role of MRP2 into bile. In turn, altered bile composition in terms of biliary load, qualitative shifts, and dilution is able to influence the trajectory of plotted points across the different zones, and the shift of zones within the ternary equilibrium bile salt–phospholipid–cholesterol phase diagram (78). The ultimate effect will be on cholesterol crystallization (Figs. 3 and 4). Animal studies confirm that pharmacologic modulation of bile flow and glutathione secretion can affect biliary composition and the risk of forming gallstones. In a murine model of gallstone disease, administration of ezetimibe increased bile flow and bile salt, phospholipid and glutathione secretion rates. These changes were linked with a moderately increased expression of hepatic bile salt transporters (73).

### Effects of bempedoic acid on Farnesoid X receptor (FXR)

FXR is a nuclear transcription factor, a critical intracellular sensor of bile acids, and a master regulator of bile acid homeostasis (79). The intracellular concentration of bile acids is constantly controlled by a negative feed-back involving the downregulation of key enzymes of bile acid synthesis and influx (15, 80, 81). These mechanisms counteract liver injury due to cytotoxicity of bile acids accumulation (14, 82, 83). FXR receptors are widely diffused in multiple organs, and FXR activation generates a variety of effects, including the regulation of gallbladder dynamics (mainly through the FGF19 signaling), the modulation of lipid, carbohydrate, and amino acid homeostasis, and the control of inflammatory pathways at hepatic and systemic levels (81). Thus, FXR dysregulation might contribute to the increased risk of cholesterol lithogenesis through effects on enterohepatic lipid and bile acid homeostasis. Likely mechanisms involve the inhibition of bile acid synthesis and excretion, the increased biliary cholesterol saturation index, the suppression of de novo lipogenesis and triglyceride accumulation, and the deranged gallbladder motility secondary to disrupted FXR/FGF19 axis (84).

In particular, in the hepatocyte, FXR activation by bile acids or by FXR agonists upregulates gene expression of *ABCG5/8*, with increased mRNA and ABCG5/ABCG8 protein expression (85, 86, 87). These mechanisms ultimately promote the efflux of hepatic cholesterol into the bile canaliculi. A similar effect in the gut controls cholesterol absorption by enterocytes (86, 87). These pathways efficiently prevent cholesterol accumulation, but also increases the cholesterol content in bile. Thus, the simultaneous absence of enough bile acids may increase the risk of cholesterol gallstones (88).

In an animal model of liver steatosis, bempedoic acid indirectly activated FXR receptors in the liver, with increased FXR transcriptional activity which resulted in quadruplicated expression of the *ABCG5* gene and inhibition of the activity of the *Cyp7a1* gene, which is also under the FXR control (57). According to this model, bempedoic acid does not directly activates FXR, and the increased transcriptional activity is instead mediated by a significant increase in the amount of Peroxisome Proliferator Activator Receptor Gamma Coactivator-1α (PGC1α) protein in the liver. This pathway reduces the ratio of phosphorylated (inactive) to total PGC1α, allowing PGC1α to coactivate FXR transcriptional activity (57). An additional mechanism is the bempedoic-mediated inhibition of the activity of liver mTORC1, which reduces the enzymatic activity of ribosomal protein S6 kinase beta-1 (S6K1), a downstream effector of mTORC1 signaling (89) responsible for PGC1α phosphorylation and inactivation (57).

In an animal model, the administration of sevelamer hydrochloride attenuated lithogenic diet-induced cholesterol gallstone formation lowering cholesterol saturation index in bile, inhibiting the intestinal FXR-FGF15 pathway, and increasing hepatic bile acid synthesis. These effects were reversed by fexaramine, a FXR agonist, which increased FGF15/Shp expression, suppressed bile acid synthesis, increased the cholesterol saturation index, and partially restored gallstone susceptibility (90).

Although the efficiency of FXR receptor stimulation induced by bempedoic acid has not been explored so far, available information could suggest a critical role for bempedoic acid in modulating the bile acid/FXR/FGF19 axis, potentially increasing bile lithogenicity and susceptibility to cholesterol gallstones.

### Effects of bempedoic acid on SLC13A5 and Pregnane X receptor (PXR)

The plasma membrane citrate transporter (SLC13A5) is a sodium-coupled transporter that mediates the cellular uptake of citrate, thus playing a critical role in the synthesis of fatty acids and cholesterol (91). SLC13A5 imports cytosolic citrate from the extracellular milieu before its use in ACLY to generate acetyl-CoA. This transporter is also a transcriptional target of the nuclear receptor PXR. When activated, PXR upregulates the expression of SLC13A5 (92). The nuclear receptor PXR also regulates the expression of genes critically involved in bile acid homeostasis, as *CYP7A1* and *Oatp2* (93). In particular, PXR activation inhibits *CYP7A1* activity (94). In mice, bempedoic acid counteracts an upregulated SLC13A5-ACLY pathway, decreasing SLC13A5 activity by suppressing PXR activation. The PXR-mediated effect of bempedoic acid has been confirmed using PXR knockout mice (95). Further studies are urged to explore if the PXR-suppressing role of bempedoic acid is secondary to direct or indirect mechanisms. In addition, the translational value of this finding needs confirmation, due to significant differences in the ligand specificity for mouse or human PXR (96). Of note, however, in the cited study the effect of bempedoic acid on the transcription of SLC13A5 and ACLY has been documented either in primary mouse hepatocytes and in human HepG2 cells (95). Thus, in humans, the suppressed PXR activity induced by bempedoic acid could play a role in increasing the risk of cholesterol gallstones by decreasing *CYP7A1* activity (i.e., decreased bile acid synthesis) and increasing the cholesterol saturation index in gallbladder bile. As shown in an animal model, PXR-null (PXR−/−) mice on lithogenic diet had reduced expression of cholesterol 7α-hydroxylase, decreased biliary concentrations of bile salts and phospholipids, and increased cholesterol saturation index. These changes promoted the formation of cholesterol crystals. In the same model, the administration of PXR agonists prevented biliary cholesterol precipitation by increasing biliary bile salt concentration and decreasing cholesterol saturation index (97).

## Translational Aspects

Specific and direct studies on the effects of bempedoic acid on factors involved in the pathogenesis of cholesterol gallstones in humans are lacking, so far. However, evidence from experimental studies indicates that bempedoic acid is able to increase the expression of *ABCG5* and *ABCG8* genes (47) (i.e., increased cholesterol efflux), to inhibit the activity of the *Cyp7a1* gene (57) (i.e., decreased bile acid synthesis), to inhibit OATPs (63, 64) (i.e., inhibited bile acid transport and entero-hepatic circulation of bile acids), and MRP1 (63) (i.e., modulation of glutathione efflux into blood, potential impact on hepatic bile concentration), to suppress PXR activity (95) (i.e., decreased bile acid synthesis, increased biliary cholesterol saturation), and to increase FXR transcriptional activity (57) (i.e., multi-level effects on bile acid/lipid homeostasis, gallbladder motility, and bile lithogenicity) (Table 1).

**Table 1:** Experimental evidence pointing to potential effects of bempedoic acid on bile lithogenicity

Model	Dose of Bempedoic Acid	Findings	Outcome	Reference
Ldlr(−/−) Mice	0, 3, 10 and 30 mg/kg body weight/day for 12 weeks	Increased expression of *ABCG5* and *ABCG8*	Increased cholesterol synthesis and secretion	(47)
Female Sprague-Dawley Rats	30 mg/kg/day for 1 month	Decreased expression of *Cyp7a1*	Decreased bile acid synthesis and secretion	(57)
Female Sprague-Dawley Rats	30 mg/kg/day for 1 month	Increased FXR transcriptional activity in the liver	Increased cholesterol synthesis and secretion	(57)
Male C57BL/6J mice	10 mg/kg/day or 30 mg/kg/day for 6 weeks	Decreased SLC13A5 activity by suppressing PXR activation	Decreased *Cyp7a1*-mediated bile acid synthesis and secretion	(95)
*In vitro* studies	-	Inhibitory effect on OATP1B1, OATP1B3 transporters	Decreased hepatic uptake of bile acids	(64)
In silico analyses	-	Inhibitory effect on MRP1	Altered transport of glutathione Altered hepatic bile concentration Increased biliary oxidative stress	(63)

Legend Ldlr(−/−), LDL receptor knockout model; *ABCG5*/8, ATP-binding cassette sub-family G member five-eighths; *Cyp7a1,* Cholesterol 7α-hydroxylase; FXR, Farnesoid X receptor; SLC13A5, Solute carrier family 13 (sodium-dependent citrate transporter), member 5; PXR Pregnane X receptor; OATP, Organic Anion Transporting Polypeptide; OAT, Organic Anion Transporter; MRP1, Multidrug Resistance-associated Protein 1.

Each mechanism needs further confirmation since, taken alone, it might not be sufficient to increase the overall risk of cholesterol lithogenesis, likely due to a number of compensatory mechanisms designed to balance bile acid homeostasis, bile composition, and biliary cholesterol saturation index.

**Fig. 5:**
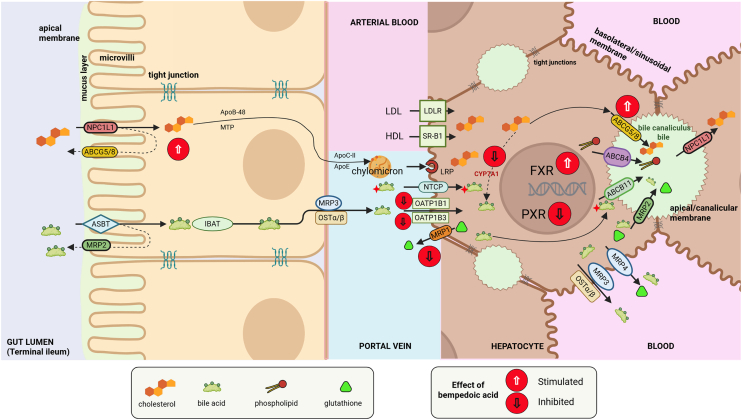
Gut-liver transport systems and pathways involved in the potential lithogenic effects of bempedoic acid. The cartoon connects the transport of cholesterol, bile acids (conjugates linked to the red star), phospholipids, and glutathione at the level of enterocyte (in the terminal ileum), portal vein, basolateral side of the hepatocyte, and bile canaliculus. Transport of lipid LDL-cholesterol and HDL-cholesterol from arterial blood is also shown. See text for details. The potential effects of bempedoic acid involve several pathways, which include increased function (red circles, white arrow ↑) and decreased function (red circles, black arrow ↓) at the level of transporter in enterocyte/hepatocytes, and nuclear receptors in hepatocytes. In synthesis, bempedoic acid is able to increase the expression of *ABCG5* and *ABCG8* genes (i.e., increased cholesterol efflux via the transporter ABCG5/8 in the canalicular space), to inhibit the activity of the *Cyp7a1* gene (i.e., decreased bile acid synthesis due to reduced function of the rate-limiting enzyme CY7A1 leading to bile acid synthesis), to inhibit bile acid transport (inhibition of bile salts uptake from portal blood flow through OATP1B1 and OATP1B3), to inhibit MRP1 (i.e., inhibition of bile acid-independent bile flow and of glutathione efflux at the basolateral membrane), to increase FXR transcriptional activity and to suppress PXR activation, with multi-level effects on bile acid/lipid homeostasis, gallbladder motility, and bile lithogenicity. Legend: NPC1L1, Niemann-Pick C1-Like 1; *ABCG5*/8, ATP-binding cassette sub-family G member five-eighths; *Cyp7a1,* Cholesterol 7α-hydroxylase; FXR, Farnesoid X receptor; PXR, Pregnane X receptor; OATP, Organic Anion Transporting Polypeptide; OAT, Organic Anion Transporter; MRP, Multidrug Resistance-associated Protein; LRP, The multifunctional, α_2_-macroglobulin/LDL receptor-related protein (LRP) is the chylomicron remnant receptor. Created in https://BioRender.com.

However, the increased incidence rate of gallstones in individuals treated with bempedoic acid (7, 9) could be in line with experimental evidence pointing to combined and multi-level effects on complex pathways governing biliary cholesterol secretion, bile acid synthesis, secretion and entero-hepatic circulation, lipid homeostasis, gallbladder motility, bile flow and biliary oxidative balance (Fig. 5).

These effects could likely operate on a background of pre-existing and well-known risk factors for cholesterol gallstones as advanced age, female gender, overweight or obesity, and other metabolic/dietary factors, associated diseases and drugs (98). In this respect, in the CLEAR Outcomes trial (7), more than half of enrolled subjects had one or more risk factors for cholesterol gallstones. The rates of individuals with ≥65 years, of female gender and/or with diabetes were 59.1%, 48.1% and 45%, respectively, and the average body mass index in the bempedoic group was 29.9 ± 5.2 kg/m^2^ (7).

In addition, other lipid-lowering therapies were permitted, such as ezetimibe, niacin, bile acid resins, fibrates, or proprotein convertase subtilisin–kexin type 9 (PCSK9) inhibitors, administered as monotherapy or in combinations.

Although only a minority of subjects were treated with fibrates (5.3% and 5.6% in the bempedoic and placebo groups, respectively) (7), according to previous evidence, this pharmacological treatment could further increase the gallstone risk (99, 100). On the other hand, experimental evidence suggests that a decreased risk could derive from combined treatments with ezetimibe (73, 101, 102) or PCSK9 inhibitors (103). Despite constitutional and pharmacological risk factors were balanced in the bempedoic and in the placebo group of the CLEAR Outcomes study, multiple possible interplays between these factors and the pharmacological effects of bempedoic acid could amplify the modulation of pathogenic pathways involved in cholesterol lithogenesis. In addition, when looking at the modest inhibitory effect of bempedoic acid on OATP1B1/3, it is possible that the pro-lithogenic effect could deteriorate during co-treatment with other drugs with similar side effects (7). Fibrates (i.e., the previously used clofibrate or currently used gemfibrozil and fenofibrate), peroxisome proliferator-activated receptor α (PPARα) agonists (100), might represent an example. These drugs increase biliary cholesterol secretion and saturation, and decrease bile acid synthesis without change of biliary phospholipid concentration (104), paving the way to manifestations of gallbladder disease (105).

A further area of concern is rapid weight loss (>1.5 kg/week), a major risk factor for cholesterol gallstones and their complications, either after a very-low-calorie diet (<800 kcal/day) or after bariatric/metabolic surgery (106, 107, 108, 109, 110, 111, 112). In this case, the increased gallstone risk mainly depends on biliary cholesterol supersaturation, increased mucin production (113, 114), and gallbladder hypomotility (115). Even after postoperative improvements in dyslipidemia, some patients will continue to meet criteria for hypolipidemic therapy, in particular if at high risk for cardiovascular events. As shown by a large matched cohort analysis, 2.6% of subjects with a previous metabolic and bariatric surgery received a new statin prescription during two years, and previous statin therapy was continued in the 36.3% of subjects after surgery (116). Based on available preclinical evidence, future studies are needed to evaluate if long-term therapy with bempedoic acid might further increase gallstone risk in subjects with a pro-lithogenic state generated by bariatric surgery.

In addition, glucagon-like peptide-1 receptor agonists (GLP-1RAs) are widely employed in the management of type 2 diabetes and obesity, with increasing prescription trends (117). The use of these pharmacological agents has been linked with increased gallstone risk (118, 119, 120), mainly due to their effects on cholecystokinin (CCK) incretion and gallbladder motility. Effects include delayed gastric emptying and deranged gallbladder motility (121), suppression of CCK release (122), decreased postprandial FGF19 and GLP-2 concentrations and increased postprandial CCK concentration, with delayed postprandial gallbladder refilling (123). The future combined use of GLP-1 RAs and bempedoic acid seems likely, either considering their increasing prescribing trend and preliminary evidence from animal studies documenting additive beneficial effects of the GLP-1 receptor agonist liraglutide and bempedoic acid on liver steatosis, ballooning and fibrosis (124). Thus, specific longitudinal studies need to explore the burden of gallstone risk and possible pathogenic pathways leading to gallstone formation in individuals treated with bempedoic acid and GLP-1 RAs, in particular if affected by chronic metabolic diseases at risk for cholelithiasis.

## Conclusions

Clues deriving from the CLEAR outcome study and from experimental evidence document the potential role of bempedoic acid in increasing the risk of cholesterol gallstones. This topic should certainly be better explored with specific future lines of research, in particular in subjects with previous risk factors for the development of cholelithiasis or during pharmacological treatments possibly linked with increased gallstone risk. Results will allow a better knowledge of the interplay between bempedoic acid and mechanisms possibly involved in the pathogenesis of cholesterol gallstones, and a safe management of this precious and effective drug in individuals at increased risk for gallstone disease.

## Conflict of interest

The authors declare that they have no conflicts of interest with the contents of this article.
